# Supplementary material for: Inclusion Complexes of Non-Steroidal Anti-Inflammatory Drugs with Cyclodextrins: A Systematic Review
Source: Biomolecules. 2021 Feb 27;11(3):361. doi: 10.3390/biom11030361 (PMC7996898; doi:10.3390/biom11030361)
Supplement: Supplementary file 1 [file biomolecules-11-00361-s001.pdf]

Supplementary Material

Table 1. Frequencies for Publication Year (2010/01/01 to 2020/02/05)

Publication Year	Frequency	Percent
2010	8	10.000
2011	10	12.500
2012	6	7.500
2013	9	11.250
2014	5	6.250
2015	5	6.250
2016	11	13.750
2017	9	11.250
2018	6	7.500
2019	6	7.500
2020	5	6.250
Total	80	100.000

Figure 1. Publication Year (2010/01/01 to 2020/02/05)

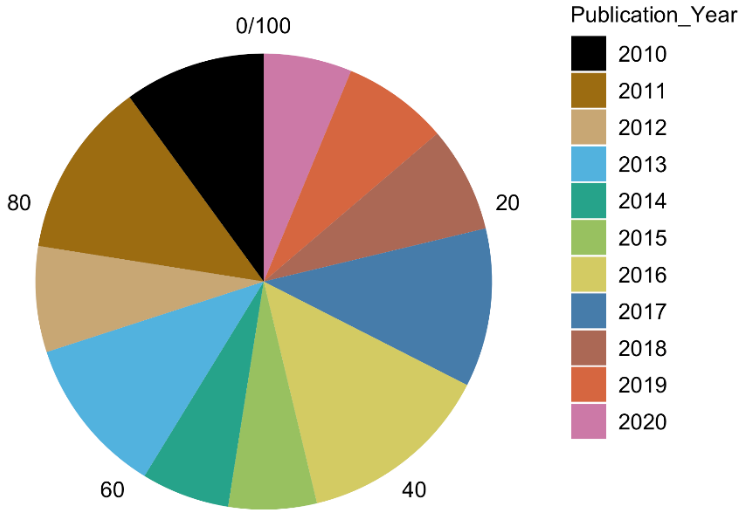

Table 2. Frequencies for NSAID

NSAID	Frequency	Percent
4-Aminosalicylic acid	1	1.176
Aceclofenac	6	7.059
Carprofen	1	1.176

Table 2. Frequencies for NSAID

NSAID	Frequency	Percent
Celecoxib	4	4.706
Dexibuprofen	2	2.353
Diclofenac	8	9.412
Etodolac	2	2.353
Etoricoxib	1	1.176
Fenoprofen	1	1.176
Flufenamic Acid	1	1.176
Flurbiprofen	8	9.412
Ibuprofen	7	8.235
Indomethacin	4	4.706
Ketoprofen	3	3.529
Lornoxicam	3	3.529
Meloxicam	11	12.941
Mesalazine	1	1.176
Naproxen	2	2.353
Nepafenac	3	3.529
Nimesulide	1	1.176
Oxaprozin	6	7.059
Piroxicam	7	8.235
Sulindac	1	1.176
Zaltoprofen	1	1.176
Missing	0	0.000
Total	85	100.000

Table 3. Frequencies for Cyclodextrin

Cyclodextrin	Frequency	Percent
2-HB-CD	1	0.870
DiMe-β-CD	1	0.870
EPI-CMβCD	1	0.870
EPI-βCD	1	0.870
HPβ-CD	40	34.783
Me-β-CD	6	5.217
Rme-β-CD	5	4.348
SBE-β-CD	3	2.609
TA-β-CD	1	0.870
α-CD	3	2.609
β-CD	46	40.000
γ-CD	7	6.087
Total	115	100.000

Table 4. Frequencies for Complexation

Complexation	Frequency	Percent
4-Aminosalicylic acid/HPβ-CD	1	0.870
4-Aminosalicylic acid/α-CD	1	0.870
4-Aminosalicylic acid/β-CD	1	0.870
4-Aminosalicylic acid/γ-CD	1	0.870
Aceclofenac/HPβ-CD	3	2.609
Aceclofenac/SBE-β-CD	1	0.870
Aceclofenac/β-CD	3	2.609
Carprofen/HPβ-CD	1	0.870
Celecoxib/DiMe-β-CD	1	0.870
Celecoxib/HPβ-CD	1	0.870
Celecoxib/Rme-β-CD	1	0.870
Celecoxib/SBE-β-CD	1	0.870
Celecoxib/γ-CD	1	0.870
Dexibuprofen/β-CD	2	1.739
Diclofenac/HPβ-CD	5	4.348
Diclofenac/Me-β-CD	1	0.870
Diclofenac/α-CD	1	0.870
Diclofenac/β-CD	3	2.609
Diclofenac/γ-CD	1	0.870
Etodolac/HPβ-CD	2	1.739
Etodolac/Me-β-CD	1	0.870
Etodolac/β-CD	1	0.870
Etoricoxib/HPβ-CD	1	0.870
Etoricoxib/β-CD	1	0.870
Fenoprofen/TA-β-CD	1	0.870
Flufenamic Acid/β-CD	1	0.870
Flurbiprofen/2-HB-CD	1	0.870
Flurbiprofen/HPβ-CD	6	5.217
Flurbiprofen/SBE-β-CD	1	0.870
Flurbiprofen/β-CD	3	2.609
Ibuprofen/HPβ-CD	2	1.739
Ibuprofen/β-CD	4	3.478
Ibuprofen/γ-CD	2	1.739
Indomethacin/HPβ-CD	3	2.609
Indomethacin/α-CD	1	0.870
Indomethacin/β-CD	1	0.870
Indomethacin/γ-CD	1	0.870
Ketoprofen/EPI-CMβCD	1	0.870
Ketoprofen/EPI-βCD	1	0.870
Ketoprofen/HPβ-CD	1	0.870
Ketoprofen/β-CD	2	1.739
Lornoxicam/HPβ- CD	1	0.870
Lornoxicam/β-CD	3	2.609

Table 4. Frequencies for Complexation

Complexation	Frequency	Percent
Meloxicam/HPβ-CD	3	2.609
Meloxicam/β-CD	9	7.826
Mesalazine/HPβ-CD	1	0.870
Naproxen/HPβ-CD	1	0.870
Naproxen/β-CD	2	1.739
Nepafenac/HPβ-CD	3	2.609
Nepafenac/γ-CD	1	0.870
Nimesulide/Me-β-CD	1	0.870
Oxaprozin/Me-β-CD	2	1.739
Oxaprozin/Rme-β-CD	4	3.478
Oxaprozin/β-CD	1	0.870
Piroxicam/HPβ-CD	4	3.478
Piroxicam/Me-β-CD	1	0.870
Piroxicam/β-CD	7	6.087
Sulindac/β-CD	1	0.870
Zaltoprofen/HPβ-CD	1	0.870
Zaltoprofen/β-CD	1	0.870
Total	115	100.000
